# Modeling the growth of *Listeria monocytogenes* on the surface of smear- or mold-ripened cheese

**DOI:** 10.3389/fcimb.2014.00090

**Published:** 2014-07-03

**Authors:** M. Sol Schvartzman, Ursula Gonzalez-Barron, Francis Butler, Kieran Jordan

**Affiliations:** ^1^Food Safety Department, Teagasc Food Research CentreMoorepark, Fermoy, Ireland; ^2^Biosystems Engineering, School of Agriculture, Food Science and Veterinary Medicine, University College DublinDublin, Ireland

**Keywords:** *Listeria monocytogenes*, growth, mathematical modeling, Baranyi and Roberts, Gompertz, Presser

## Abstract

Surface-ripened cheeses are matured by means of manual or mechanical technologies posing a risk of cross-contamination, if any cheeses are contaminated with *Listeria monocytogenes*. In predictive microbiology, primary models are used to describe microbial responses, such as growth rate over time and secondary models explain how those responses change with environmental factors. In this way, primary models were used to assess the growth rate of *L. monocytogenes* during ripening of the cheeses and the secondary models to test how much the growth rate was affected by either the pH and/or the water activity (a_w_) of the cheeses. The two models combined can be used to predict outcomes. The purpose of these experiments was to test three primary (the modified Gompertz equation, the Baranyi and Roberts model, and the Logistic model) and three secondary (the Cardinal model, the Ratowski model, and the Presser model) mathematical models in order to define which combination of models would best predict the growth of *L. monocytogenes* on the surface of artificially contaminated surface-ripened cheeses. Growth on the surface of the cheese was assessed and modeled. The primary models were firstly fitted to the data and the effects of pH and a_w_ on the growth rate (μ_max_) were incorporated and assessed one by one with the secondary models. The Logistic primary model by itself did not show a better fit of the data among the other primary models tested, but the inclusion of the Cardinal secondary model improved the final fit. The a_w_ was not related to the growth of *Listeria*. This study suggests that surface-ripened cheese should be separately regulated within EU microbiological food legislation and results expressed as counts per surface area rather than per gram.

## Introduction

Smear-ripened and mold-ripened cheeses are soft or semi-soft cheeses, matured by means of a microflora spread on the cheese surface. The maturation process in the presence of this microflora develops the characteristic aroma, flavor, and texture of these cheeses. In terms of food safety, manual or mechanical smearing technologies are associated with the risk of cross-contamination either from the processing environment or from other products (Tenenhaus-Aziza et al., 2013). *Listeria monocytogenes* can persist and survive in soft and semi-soft cheeses (Jason, 1983; Hicks and Lund, 1991; Morgan et al., 2001; Chambel et al., 2006) as well as in the cheese processing environment (Silva et al., 2002; Fox et al., 2011). Rudolf and Scherer (2001) reported in a study conducted on several European countries that 15.8% of smear cheeses contained organisms of the genus *Listeria*, confirming that contamination within dairy plants was persistent over a period of several weeks to months, that cross-contamination was an important factor contributing to this persistence and that *L. monocytogenes* can survive in raw or pasteurized milk cheeses (Dalmasso and Jordan, 2014).

*Listeria monocytogenes* is a foodborne pathogen widely distributed in nature. It is the causative agent of listeriosis when consumed and is especially associated with ready-to-eat foods such as soft or semi-soft cheese. Food safety authorities aim to regulate the occurrence of this pathogen in foods due to its 30% mortality rate in certain risk groups (pregnant, neonatal, elderly, and people with their immune system compromised) (Swaminathan and Gerner-Smidt, 2007). In 2006, listeriosis was reported in 23 EU Member States and was the fifth most common zoonotic infection in Europe. Listeriosis carries one of the highest hospitalization rates among known foodborne pathogens (91%) (Denny and McLauchlin, 2008; Scallan et al., 2011) and requires enhanced surveillance which involves elevated costs derived from excluding or treating the disease. In line with these facts, simply put, EU legislation on microbiological criteria of foods has established that there should be absence of this organism in food if the food supports growth of the organism and <100 cfu/g in foods not supporting growth during the shelf-life (European-Commission, 2005). In fact, the legislation is more complex that this, and should be consulted for greater detail.

Mathematical models in food predictive microbiology are used to describe the behavior of microorganisms under different physicochemical conditions such as pH, temperature or water activity (a_w_), and are termed “deterministic models.” These models allow the prediction of microbial safety or shelf life of products and facilitate detection of critical points on the production and distribution process (Zwietering et al., 1991). In deterministic modeling, the procedure usually consists of developing a primary model which measures the responses of interest over time, for example, maximum specific growth rate, lag phase duration, time to reach a specified level (cell numbers or metabolites) or death rate. A secondary model is then coupled to the primary model to show the dependency of these factors on environmental conditions (McMeekin and Ross, 2002; Fakruddin et al., 2011). For example, with a primary model one can estimate the growth rate at three different pH values. The primary model will provide the growth rate for each pH tested but if it is required to quantify how much the growth rate changes when the pH changes, then a secondary model is needed. The two models coupled will allow the user to predict the growth rate at any pH value within the maximum and minimum experimental values used with the primary model, even if those conditions where not tested. In this way, mathematical models predicting the growth rate and final concentration of *L. monocytogenes* in cheese can help evaluate Food Business Objectives taking into account the inherent processing and variability in cheese conditions. Many models are based on data obtained from liquid microbiological media and are used to describe the possible impact of several factors (e.g., pH, a_w_, organic acids concentrations) when these values are known (Anonymous, 2008). Behavior of bacteria in liquid laboratory media does not always reflect the reality of behavior of bacteria in food systems (Schvartzman et al., 2010).

Different approaches can be used to modeling microorganisms in food. Spor et al. (2010) used a logistic equation in a Bayesian framework to model population dynamics of *Saccharomyces cerevisiae*, Delignette-Muller et al. (2006) used Bayesian modeling to study growth of *L. monocytogenes* in cold-smoked salmon and Barker et al. (2005) used a probabilistic approach to understanding consumer exposure to *Clostridium botulinum* neurotoxin in minimally processed foods. These different approaches could be applied to understanding growth of *L. monocytogenes* in cheese. In addition, McMeekin et al. (1993), provides an excellent review and discussion of the classical sigmoidal growth functions, especially the Logistic and Gompertz equations. The models chosen for this study have been used extensively by researchers to model the growth of pathogens in food (Gibson et al., 1987; Alavi et al., 1999; Castillejo Rodríguez et al., 2000; Bovill et al., 2001; Gospavic et al., 2008; Spor et al., 2010) and to assess microbial growth parameters in risk assessment studies (Pouillot et al., 2003; Delignette-Muller et al., 2006).

In this study, challenge tests were carried out by spiking *L. monocytogenes* onto the surface of commercially acquired cheeses just after manufacturing and prior to ripening. With the data obtained, three primary and three secondary models were tested in order to define which combination of primary and secondary models would best predict the behavior of *L. monocytogenes* on the surface of smear-ripened and mold-ripened cheeses, based on a_w_ and pH measurements.

## Materials and methods

### Culture preparation

The two strains used (strain 6179 serotype 1/2a and strain C5 serotype 4b) are natural isolates from cheese and the environment, respectively. Stocks of *L. monocytogenes* were kept in Tryptic Soy Broth (TSB; Becton Dickinson Co., USA) and glycerol at −80°C. For each experiment, cultures were prepared by two consecutive overnight growths in TSB at 37°C. The cultures were mixed and diluted in maximum recovery diluent (MRD, Oxoid, Basingstoke, UK) for each experiment to achieve the desired inoculum level of 10^3^ cfu/ml of inoculation solution.

### Experimental design

Challenge tests were performed in 4 batches.

- batch 1: 20 smear-ripened cheeses ripened for 30 days (not washed)
- batch 2: 20 smear-ripened cheeses ripened for 30 days (not washed)–a replicate of batch 1 undertaken with cheese manufactured on a different day
- batch 3: 16 mold-ripened cheeses ripened for 12 days (not washed)
- batch 4: 10 smear-ripened cheeses ripened for 25 days (washed)–a replicate of batch 1 undertaken with cheese manufactured on a different day

### Inoculation, ripening, and sampling

#### Smear-ripened cheeses

Forty raw milk cheeses (20 from batch 1 and 20 from batch 2) were obtained from the producer following manufacture. Batch 1 and batch 2 were independent experiments where cheeses had already been smeared by the producer with a culture-mix of bacteria and yeasts. Each cheese unit was spiked with *L. monocytogenes* by shaking them in a sterile plastic bag with 100 ml of *L. monocytogenes* solution (10^3^ cfu/ml), they were then left to drain for 10 min and placed in an incubator at 15°C and 90% relative humidity for 15 days and at 7°C for a further 15 days, according to the technology of the producer. These cheeses were not surface-washed throughout the ripening period. Four cheeses (two from each batch) were sampled in duplicate every 3–4 days and pH, a_w_, and *Listeria* counts were determined at each sampling. The pH was measured with an Orion pH meter model 420A, the British Standard for pH determination of cheese samples was followed (BS770:5:1976). The a_w_ was measured in an AquaLab Series 3T equipment (Labcell, Hampshire, UK) by sampling a portion of cheese as described in the manufacturers instructions. For the surface counts of *L. monocytogenes*, 4 cm^2^ (2 × 2 cm wide by 1 mm depth) were sampled from the surface of each cheese following the sampling and initial dilution of cheese procedure for microbiological analysis (IDF 50B:1985 and 122B:1992). Cell numbers were estimated by the spread plate method using Agosti and Ottaviani Listeria Agar and expressed in units of area, cfu/cm^2^. The growth curves obtained were used for model fitting.

#### Mold-ripened cheese

Sixteen pasteurized milk cheeses (batch 3) were acquired from the producer ready to be ripened. They were spiked as described above and placed in individual plastic boxes with a saturated salt solution of potassium sulfate for humidity control. Each box was then placed in an incubator at 12°C for 12 days, as indicated by the producer. Four cheeses were sampled every 4 days. Analyses for pH, a_w_, and *Listeria* were carried out as described above. The data obtained were used for model fitting.

#### Data for model validation

Ten cheeses (batch 4) were acquired from the producer, inoculated with *L. monocytogenes* and ripened as indicated for smear-ripened cheeses. During ripening, cheeses were washed every 3 days with sterile saline water (5% NaCl) by rubbing the cheeses with a sterile brush in order to eliminate the adventitious mold that grew on the surface; two cheeses were sampled every 3–4 days and pH, a_w_, and *Listeria* counts were determined for each sampling; this procedure was adopted in order to determine whether the brushings with saline solution would affect the numbers of *L. monocytogenes* growing on the surface. The data obtained from this batch of cheeses were used for comparison with growth observed in “not washed” cheeses and as an independent set of data for validation of the model.

### Modeling

#### Primary models

In order to estimate first order kinetic parameters such as the maximum specific growth rate, lag phase duration and the time to reach a maximum concentration, three commonly used primary models were tested in this study, namely, the modified Gompertz equation (Xiong et al., 1999), the Baranyi and Roberts model (Baranyi and Roberts, 1994), and the Logistic Model (Causton, 1977; Jason, 1983).

The modified Gompertz equation used was of the following form:

$$N(t)=N(0)+Ce^{-e^{-B(t-M)}}$$

Where *N(t)* is the total population at time *t* in log cfu/cm^2^, *N*(0) is the population at time 0 in log cfu/cm^2^, *B* the relative growth rate at *M* (h^−1^), *C* is the difference in value between the higher and lower asymptote, and *M* is the time at which the absolute growth rate is maximal (h). The model has three parameters to be estimated: *C*, *B*, and *M*.

The explicit form of the Baranyi model used was the following (Baranyi and Roberts, 1994; Grijspeerdt and Vanrolleghem, 1999):

$$N(t)=N(0)+\mu_{\max}\cdot t+\frac{1}{\mu_{\max}}\cdot Ln\left(e^{-vt}+e^{-h_{0}}-e^{-vt\;-\;h_{0}}\right)-\frac{1}{m}\cdot Ln\left(1+\frac{e^{m\cdot \mu_{\max}\cdot t\;+\;\frac{1}{\mu_{\max}}\cdot Ln(e^{-vt}\;+\;e^{-h_{0}}\;-\;e^{-vt-h_{0}})}}{e^{m\cdot (N_{\max}\;-\;N(0))}}\right)$$

Where *N(t)* is the total population in log cfu/cm^2^, *N*(0) is the initial population in log cfu/cm^2^, μ_max_ is the maximum specific growth rate (h^−1^), *m* is a curvature parameter to characterize the transition from the exponential phase, *v* is a curvature parameter to characterize the transition to the exponential phase and *h*_0_ is a dimensionless parameter quantifying the initial physiological state of the cells. The *h*_0_ parameter could be estimated from the fitting process or calculated as $h_{0}=\frac{\mu_{\max}}{\lambda }$ being λ the lag time. Since the curves presented a lag phase in only some cases, the *h*_0_ parameter was set to 0 when no lag phase was observed; and 2 when there was a lag phase.

For the curvature parameters, Baranyi (1997) suggested that *v* = μ_max_ and *m* = 1. These values have been assumed in this work as well. The model then adopts the following form:

$$N(t)=N(0)+X-Ln\left(1+\frac{e^{X}}{e^{\left(N_{\max}-N(0)\right)}}\right)X=\mu_{\max}\cdot t+\frac{1}{\mu_{\max}}\cdot Ln\left(e^{-\mu_{\max}\cdot t}+e^{-h_{0}}-e^{-\mu_{\max}\cdot t\;-\;h_{0}}\right)$$

given that *h*_0_ could be calculated as $\frac{\mu_{\max}}{\lambda }$ (λ = lag time), cases where no lag was observed, λ = 0 and consequently *h*_0_ = 0. The model has now three parameters to be estimated: μ_max_; *h*_0_ and *N*_max_.

The Logistic model used is an integrated form of the original Verhulst model (Causton, 1977; Jason, 1983):

$$\frac{\partial N}{\partial t}=\mu N\cdot \left(1-\frac{N}{N_{\max}}\right)$$

The equation assumes that the starting position (*t* = 0) equates to the beginning of the sigmoidal curve (Vose, 2008). The explicit form of the equation used was:

$$N(t)=\frac{N_{\max}\cdot N(0)\cdot e^{\left(\mu_{\max}\cdot t\right)}}{N_{\max}+N(0)\cdot \left[e^{\;(\mu_{\max}\cdot t)}-1\right]}$$

Where *N*(*t*) is total population in log cfu/cm^2^, *N*_max_ is maximum bacterial concentration in log cfu/cm^2^, *N*(0) is initial bacterial concentration (log cfu/cm^2^) and μ_max_ is maximum specific growth rate (h^−1^). The logistic model has two parameters to be estimated: *N*_max_ and μ_max_.

#### Secondary models

To show the dependency of the parameters defined with the primary models on environmental conditions, a simple model was assumed with the following form: μ_max_ = μ_opt_ · *X*(pH) · *X*(a_w_) where μ_opt_ is the optimum growth rate, i.e., the maximum growth rate at optimum pH or a_w_ conditions, and *X*(pH) *and X*(*a*_w_) are functions of pH and a_w_, respectively. The functions for pH and a_w_ used are summarized in Table 1. These models have been previously tested by Augustin et al. (2005) in an evaluation study of growth rates and growth probabilities of *L. monocytogenes* in dairy and other foods under suboptimal conditions. In these models, pH_max_ is the nominal value of pH above which no growth occurs, pH_min_ or a_wmin_ are the nominal values of pH or a_w_ below which no growth occurs, and pH_opt_ or a_wopt_ correspond to the values of pH or a_w_ at which the μ_max_ is optimal.

**Table 1:** **pH and a_w_ functions [*X*(pH) and *X*(a_w_)] evaluated in the secondary Cardinal model (CM), Ratkowsky model (RM), and Presser model (PM)**.

**Model**	**Equations**	**Effect on *μ_**max**_***
CM	$$CM(\text{pH})=\{\begin{array}{l} 0,\text{ pH}\leq {\text{pH}}_{\min}\\ \frac{\left(\text{pH}-{\text{pH}}_{\min}\right)\left(\text{pH}-{\text{pH}}_{\max}\right)}{\left({\text{pH}}_{\text{opt}}-{\text{pH}}_{\min}\right)\left(\text{pH}-{\text{pH}}_{\text{opt}}\right)-\left({\text{pH}}_{\text{opt}}-{\text{pH}}_{\max}\right)\left({\text{pH}}_{\min}-\text{pH}\right)},\;{\text{pH}}_{\min}<\text{pH}<{\text{pH}}_{\max} \end{array}$$	pH
RM	$$RM(X)=\{\begin{array}{l} 0,\;X\leq X_{\min}\\ \left(\frac{\left(X-X_{\min}\right)}{\left(X_{\text{opt}}-X_{\min}\right)}\right),\;X_{\min}<X<X_{\text{opt}} \end{array}$$	pH and a_w_
PM	$$PM(\text{pH})=\{\begin{array}{l} 0,\;X\leq X_{\min}\\ \frac{1-10^{{\text{pH}}_{\min}\;-\text{ pH}}}{1-10^{{\text{pH}}_{\min}\;-\;{\text{pH}}_{\text{opt}}}},X_{\min}<X\leq X_{\text{opt}} \end{array}$$	pH

Secondary models express the variability of the μ_max_ as a function of the environmental parameters (pH and a_w_), reflection of metabolic activity and influence of *in situ* conditions. The cardinal model (CM) was developed by Rosso et al. (1995) and is known as the cardinal model because it contains cardinal values for the growth of the organisms in question, namely, maximum, minimum, and optimum values for growth. The advantage of including cardinal parameters in the model are based on the biological significance, specific for each organism, which personalizes the model to the microorganism evaluated.

The Ratkowsky model (RM) was developed by Ratkowsky et al. (1982) in an attempt to describe the effect of temperature on microbial growth. The model was a simple form explaining the square root of the growth rate (μ_max_) as the multiplication of a constant (μ_opt_) by the difference between the temperature and the temperature at time 0. [√ rate = *c*(*T* – *T*_0_)]. This model was later modified to include maximum and optimum temperatures as variables. Zwietering et al. (1991) evaluated a series of models and found the modified Ratkowsky model to be the most suitable for both growth rate and asymptote (maximum population size) as a function of temperature. Augustin et al. (2005) introduced this model to explain the effect of pH and a_w_ on the maximum growth rate.

The Presser model (PM) was proposed by Presser et al. (1997) based on the Bělehrádek-like model for the effects of a_w_ and temperature. Presser et al. (1997) expanded the model to describe the effect of pH and lactic acid. The model was developed for application to foods at suboptimal pH conditions, i.e., from acidic pH to neutral pH, on the basis that growth rate response is directly proportional to the hydrogen ion concentration [H^+^]. Tiengenun et al. (2000) later used this model in their study to describe the μ_max_ of *L. monocytogenes* in broth medium as a function of temperature, a_w_, pH, and lactic acid concentrations.

#### Modeling and goodness of fit

The primary models were firstly fitted to the data and secondly, the effects of pH and a_w_ on the growth rate (μ_max_) were incorporated and assessed one by one with the secondary models. The fitting process was carried out with SAS software version 9.1 (SAS Institute Inc., Cary, NC, USA) with the *PROC NLMIXED* procedure and the criteria used to evaluate goodness of fit among the models were the Log likelihood (Log-like), the Akaike Information Criterion (AIC) and the Bayesian Information Criterion (BIC). These three tests provide information on the quality of the statistical model. Each of the primary and secondary models were compared with the BIC value, since the Baranyi model has one extra parameter. The *F*-test was also used to establish a comparison of the variance difference between the observed and the predicted numbers of *L. monocytogenes*. An *F*-value close to 1 means that the differences between observed and predicted values was very low and thus a good fit was achieved.

## Results

### Growth of *L. monocytogenes*

**Figure 1:**
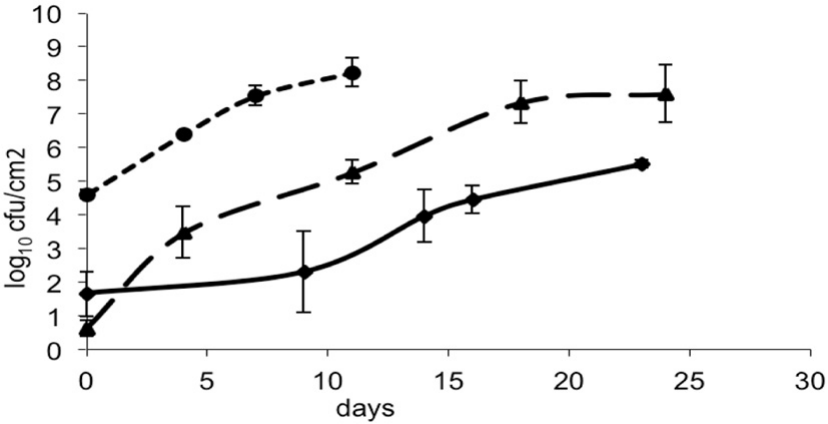
**Growth of *L. monocytogenes* in smear-ripened cheeses (—), (— —) and in mold-ripened cheeses (– – –) with error bars corresponding to four replicates**.

The bacteria- and mold-ripened cheeses tested (batches 1–3) supported the growth of *L. monocytogenes* during ripening. Three growth curves were obtained (1 for each batch) for the growth of *L. monocytogenes*, with each time point being an average of four independent replicates. Numbers of *L. monocytogenes* were estimated and expressed as log_10_ colony forming units per cm^2^ (log cfu/cm^2^). The growth observed in batch 2 had a lag phase of circa 10 days and the maximum population reached was variable, having reached maximum numbers of 8.64 log_10_ cfu/cm^2^ in batch 3. Maximum population numbers were reached between days 18 and 24 in batches 1 and 2 and at day 11 in batch 3. Curves corresponding to the growth of *L. monocytogenes* are shown in Figure 1, averages for each sampling point and standard deviations corresponding to the four independent replicates were plotted.

**Figure 2:**
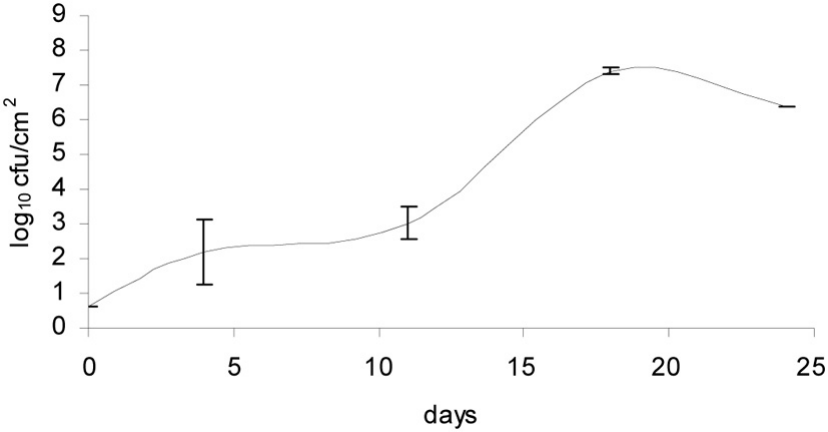
**Growth of *L. monocytogenes* in smear-ripened cheeses washed and brushed during ripening**.

Washed and brushed cheeses during ripening (batch 4) also supported the growth of *L. monocytogenes*. The growth observed in washed cheeses is shown in Figure 2. Averages for each sampling point and standard deviations corresponding to the two independent replicates were plotted.

### pH and water activity

**Figure 3:**
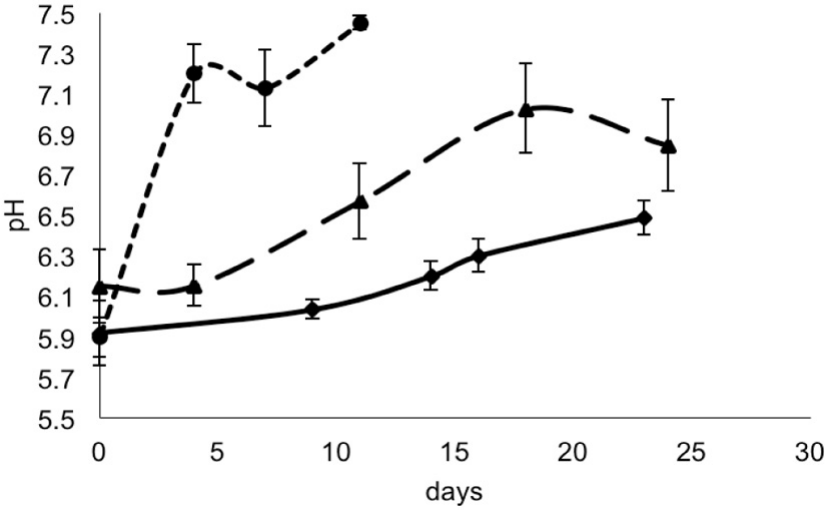
**pH in smear-ripened cheeses batch 1 (—), batch 2 (——) and in mold-ripened cheeses, batch 3 (– – –)**.

**Figure 4:**
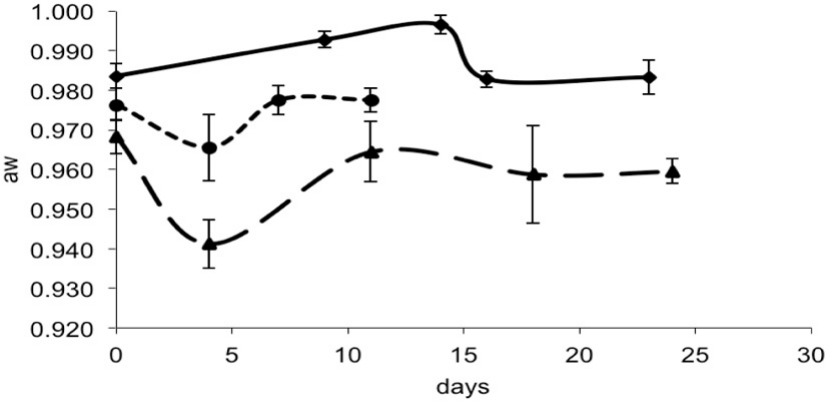
**a_w_ in smear-ripened cheeses batch 1 (—) and batch 2 (— —) and in mold-ripened cheeses batch 3 (– – –)**.

For each data point, the values of pH and a_w_ of the cheese surface were obtained (Figures 3, 4). The pH increased from 5.7 to 7.3 in 4 days in mold-ripened cheeses, whereas in bacterial ripened cheeses, the increase was smoother and lower [from 6 to 7 in 20 days (batch 1) and from 5.9 to 6.4 (batch 2)]. The a_w_ varied considerably between batches and did not show a clear trend during the ripening. Initial levels of a_w_ on the surface of cheese ranged from 0.968 to 0.988. Minimum values of a_w_ were reached in batch 2 in 5 days, corresponding with the initial lag phase observed in this batch.

### Primary model performance

**Figure 5:**
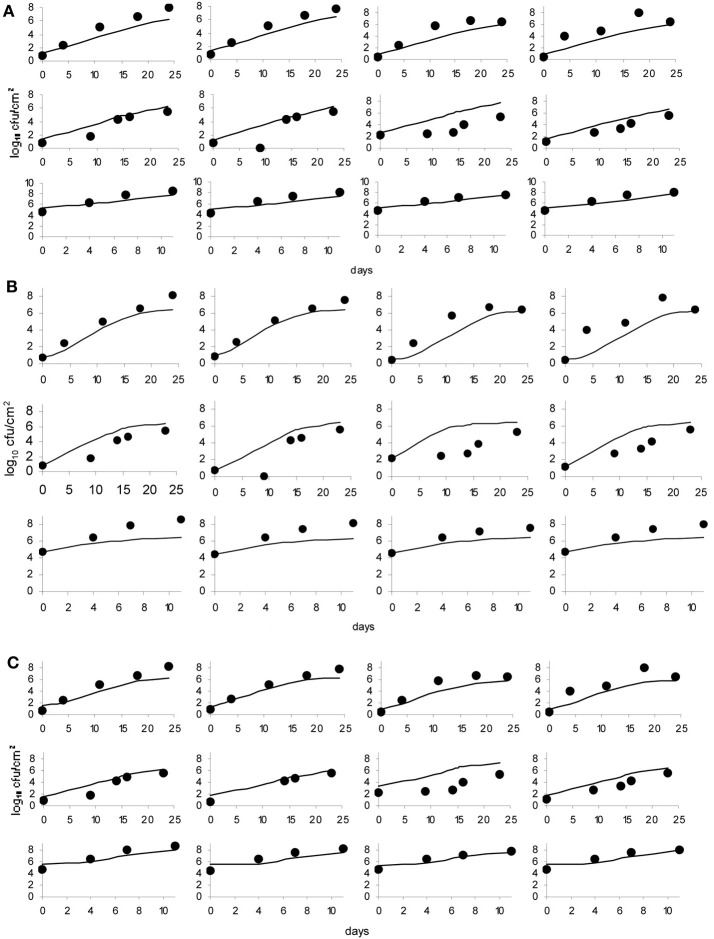
**Baranyi primary model (A), Logistic model (B) and Gompertz model (C); dots represent observed data and lines represent the predicted outcome**.

The data obtained for growth of *L. monocytogenes* on the surface of smear-ripened cheese were fitted with the primary models: modified Gompertz, modified Baranyi and Logistic model. The performance of the models was assessed with the BIC index, and with the *F*-test shown in Table 2 for each model; the Baranyi model had the lowest BIC index although the *F*-test showed worst performance. The fit of the three primary models is shown in Figure 5. Overall, the difference between the fitting indices of the primary models was very low. Table 3 shows the estimates of the primary models with their standard errors and *p*-values.

**Table 2:** **Goodness of fit of the primary models**.

**Primary models**	**LogLike**	**AIC**	**BIC**	***F*-test**
Logistic model	192	198	204	1.41
Modified Gompertz	187	195	203	1.48
Baranyi's model	183	191	199	1.51

**Table 3:** **Estimates of the primary models with their standard errors and *p*-values**.

	**Logistic**	**Gompertz**	**Baranyi**
μ_max_	0.2557	0.1026	0.4465
Standard error	0.04217	0.03618	0.03818
*p*-value	<0.0001	0.0064	<0.0001
*N*_max_	6.5323	–	6.8333
Standard error	0.4316	–	0.4701
*p*-value	<0.0001	–	<0.0001
*M*	–	9.1007	–
Standard error	–	3.0676	–
*p*-value	–	0.0044	–
*C*	–	6.9492	–
Standard error	–	1.6938	–
*p*-value	–	<0.0001	–

*μ_max_, maximum specific growth rate; N_max_, maximum population number; M, time at which the absolute growth rate is maximum; C, difference in value between the higher and lower asymptote; –, parameter not in the model*.

### Secondary model performance

The effect of pH and a_w_ on the maximum growth rate was tested with the secondary models: CM, RM, and PM. The pH was considered as a variable first and the model fit was assessed with the Log-like, AIC and BIC indices as well as with the *F*-test (Table 4). Of the three primary models, the Logistic model showed the greatest improvement of fit, when expressing μ_max_ as a function of pH with the CM.

**Table 4:** **Goodness of fit of the secondary models with pH as a variable**.

**Secondary models**	**CM**				**RM**				**PM**			
**Primary models**	**LogLike**	**AIC**	**BIC**	***F*-test**	**LogLike**	**AIC**	**BIC**	***F*-test**	**LogLike**	**AIC**	**BIC**	***F*-test**
Logistic model	144	154	157	1.11	167	175	183	0.97	166	174	182	–
Modified Gompertz	208	220	232	1.48	186	196	207	1.35	193	201	209	1.49
Baranyi's model	–	–	–	–	174	184	194	1.15	179	189	200	0.69

*–, no convergence; CM, cardinal model; RM, Ratkwosky model; PM, Presser model*.

Despite the low statistical indices obtained with the Baranyi primary model, parameters estimated with the CM were not significant either with pH, a_w_ or the combination of the two factors for the estimation of μ_max_.

The Gompertz model had a good fit with the three secondary models. In general, the fit of the Baranyi model, when possible (as there was no convergence with the CM), and the modified Gompertz primary models were not improved much by inclusion of the secondary models. Moreover, inclusion of a_w_ as a variable with the secondary model, did not improve the model performance, but made the parameters non-significant, in other words, there was a lack of fit of the models to the data when a_w_ was incorporated.

### Logistic cardinal model

The Logistic primary model was chosen to model the data of *L. monocytogenes* growth on smeared-cheese surface because its fit with the inclusion of the secondary models for pH was better than the Gompertz model. The inclusion of a_w_ did not improve the fit (Log-like 216: AIC: 224 and BIC: 232) and it was therefore not used as a variable for the model predictions. The CM had Log, AIC and BIC indices lower than the RM, but the *F*-test for the RM resulted in better values than the CM (see Table 3). Therefore, the primary and secondary models used were the logistic primary model and the CM:

$$N(t)=\frac{N_{\max}\cdot N(0)\cdot e^{\left(\mu_{\max}\cdot t\right)}}{N_{\max}+N(0)\cdot \left[e^{\left(\mu_{\max}\cdot t\right)}-1\right]}\text{being }\mu_{\max}=\mu_{\text{opt}}\cdot CM(\text{pH})$$

And

$$\text{CM}(\text{pH})=\{\begin{array}{l} 0,\text{ pH}\leq {\text{pH}}_{\min}\\ \frac{\left({\text{pH}-\text{pH}}_{\min})({\text{pH}-\text{pH}}_{\max}\right)}{\left({\text{pH}-\text{pH}}_{\min}\right)\left({\text{pH}-\text{pH}}_{\text{opt}}\right)-\left({\text{pH}}_{\text{opt}}{-\text{pH}}_{\max}\right)\left({\text{pH}}_{\min}-\text{pH}\right)},\\ \;{\text{pH}}_{\min}<\text{pH}<{\text{pH}}_{\max} \end{array}$$

**Figure 6:**
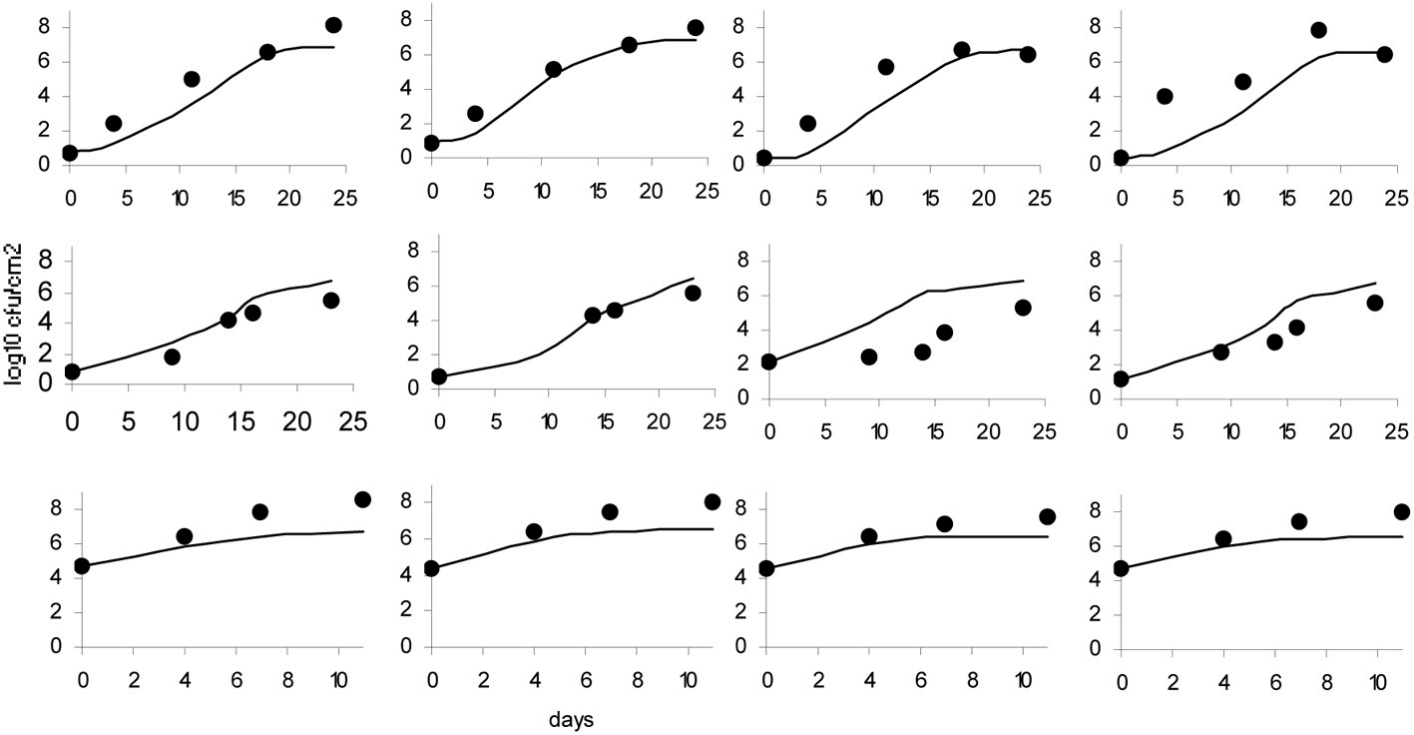
**Observed growth (•) and Logistic Cardinal model predicted growth (—)**.

The μ_opt_ value fitted by the model was 0.2835 days^−1^. *N*_max_ was 7.4272 log_10_ cfu/cm^2^ and pH_max_ was estimated by the model fit as 7.6341. The model converged for pH_min_ and pH_opt_ values of 5.2 and 7.1 respectively, which were arbitrarly set. Observed and predicted growth data for the Logistic Cardinal model were plotted in Figure 6.

### Model validation

**Figure 7:**
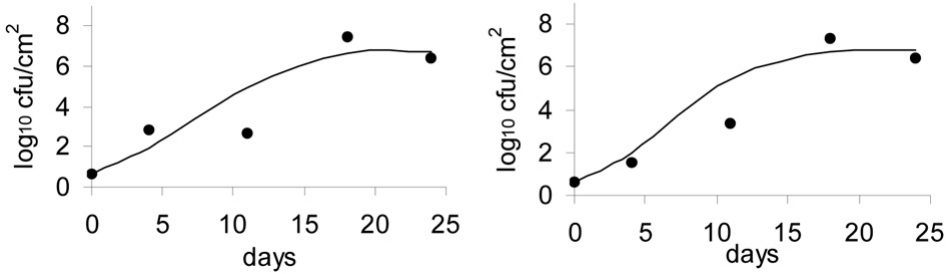
**Validation of the Logistic Cardinal model with data on growth of *L. monocytogenes* on the surface of smear-ripened cheese**.

The model was validated with an independent set of data, more precisely, the data obtained from the washed smear-ripened cheeses (batch 4). The model accurately predicted the general trend of the population growth and the final numbers. Plots of the observed against the predicted growth are shown in Figure 7.

## Discussion

In this study, the fate of *L. monocytogenes* on the surface of commercial cheese, simulating an environmental cross-contamination event, was evaluated and modeled, using the best combination of a primary and secondary models selected from the three primary and three secondary models tested (Table 4; Figure 6). *L. monocytogenes* was found to grow on the surface of the cheese during ripening of pasteurized or raw milk surface-ripened cheeses. In order to account for strain variability, two strains were used in the challenge tests; cheese variability and consequent variability in pathogen behavior was taken into account by performing the experiments in two different types of surface-ripened cheeses (bacterial and mold-ripened cheeses). It is evident from the scientific literature that surface-ripened cheeses constitute a threat to public health, showing high occurrence and persistence of *L. monocytogenes*. Other studies have suggested that *L. monocytogenes* persists in the environment (Silva et al., 2002; Chambel et al., 2006; Fox et al., 2011) and survives on the surface of, or in the core of the cheese (Jason, 1983; Ryser and Marth, 1987; Silva et al., 2002; Chambel et al., 2006). This study supports survival on the surface.

From the growth observed in all the 12 sets of data obtained with different cheeses (Figure 1), it can be deduced that the microflora present on the surface did not cause inactivation of the populations of *L. monocytogenes* by competition. The different flora present on the surface of the cheeses contributed to changes in pH (Figure 3); smear-ripened cheeses had similar pH profiles throughout ripening reaching maximum values of 6.67 (±0.24), but mold-ripened cheeses yielded higher pH values, with final maximum values of 7.45 (±0.03). These values are in accordance with other bacterial- and mold-ripened cheeses (Bockelmann and Hoppe-Seyler, 2001; Abraham et al., 2007; Liu and Puri, 2008). These high pH values probably contributed positively to the growth of *L. monocytogenes*. It can be said therefore that the presence of certain microorganisms on these cheese-types indirectly enhanced the populations of *L. monocytogenes* by increasing pH. The pH range was relatively high and was probably the factor that allowed *L. monocytogenes* populations to grow from 2.5 to 7.3 log-cycles. On the other hand, the humidity conditions of maturation together with the salt content were determined by the a_w_ measurements (Figure 4). However, the a_w_, was not related to the growth of *L. monocytogenes*, reflected in the lack of correlation between the two variables and in the lack of fit of the models by inclusion of this parameter.

The Gompertz equation has previously been used to predict the growth of *L. monocytogenes* in milk (Murphy et al., 1995) and was validated in a range of dairy products. In this study, the Gompertz model showed a good fit although the secondary models tested did not converge with the data using the Gompertz model. The Logistic primary model by itself did not show the best fit of the data among the other primary models tested, but the inclusion of the Cardinal secondary model improved the final fit. The usefulness of the combined logistical and cardinal models, called the Logistical Cardinal model, to predict growth of *L. monocytogenes* on the surface of smeared cheese was further proven with validation of the model (Figure 7). The set of data used to validate the model was obtained independently and in different conditions than the data used to create the model. The cheeses used for the validation data were washed throughout ripening by means of a brush with saline water. The data obtained showed that *L. monocytogenes* was able to grow despite the physical action applied with the brushings. This, on the other hand, demonstrates that cheeses with high surface *pH* and a_w_, present an actual elevated risk in terms of food safety, since the final number of *L. monocytogenes* on the cheese surface after 11–25 days of ripening were ~7 log cfu/cm^2^.

Current legislation controlling the safety of foods and protecting consumers states that products supporting the growth of *L. monocytogenes* should not exceed the limit of 100 cfu/g throughout their shelf life. In order to test this, a 25 g wedge of cheese would typically be diluted 10 fold, blended in a stomacher and plated onto agar plates. With this procedure, surface, and core are homogenized together and the surface contamination is therefore diluted giving a misleading picture of any actual contamination. This study suggests that this type of cheese should be contemplated in the EU regulations as higher risk products. Furthermore, this study proposes that assessment of *L. monocytogenes* contamination on the surface of cheeses should be reported separately in counts per surface area rather than per weight.

## Author contributions

M. Sol Schvartzman and Ursula Gonzalez-Barron were involved in the necessary laboratory and computer work. Kieran Jordan and Francis Butler were involved in obtaining funding and designing experiments. All authors contributed to preparing the final manuscript.

### Conflict of interest statement

The authors declare that the research was conducted in the absence of any commercial or financial relationships that could be construed as a potential conflict of interest.

## References

[B1] Abraham S. Cachon R. Colas B. Feron G. De Coninck J. (2007). Eh and pH gradients in Camembert cheese during ripening: measurements using microelectrodes and correlations with texture. Int. Dairy J. 17, 954–960 10.1016/j.idairyj.2006.12.010

[B35] Alavi S. H. Puri V. M. Knabel S. J. Mohtar R. H. Whiting R. C. (1999). Development and validation of a dynamic growth model for *Listeria monocytogenes* in fluid whole milk. J. Food Prot. 62, 170–176 10030637 10.4315/0362-028x-62.2.170

[B2] Anonymous. (2008). Guidance Document on Listeria Monocytogenes Shelf-Life Studies for Ready-to-Eat Foods, Under Regulation (EC) No 2073/2005 of 15 November 2005 on Microbiological Criteria for Foodstuffs. Brussels: Commision of the European Communities

[B3] Augustin J.-C. Zulliani V. Cornu M. Guiller L. (2005). Growth rate and growth probability of *Listeria monocytogenes* in dairy, meat and seafood products in subGimal conditions. J. Appl. Microbiol. 99, 1019–1042 10.1111/j.1365-2672.2005.02710.x 16238733

[B4] Baranyi J. (1997). Commentary: simple is good as long as it is enough. Food Microbiol. 14, 189–192 10.1006/fmic.1996.0080

[B5] Baranyi J. Roberts T. A. (1994). A dynamic approach to predicting microbial growth in food. Int. J. Food Microbiol. 23, 277–294 10.1016/0168-1605(94)90157-0 7873331

[B36] Barker G. C. Malakar P. K. Del Torre M. Stecchini M. L. Peck M. W. (2005). Probabilistic representation of the exposure of consumers to *Clostridium botulinum* neurotoxin in a minimally processed potato product. Int. J. Food Microbiol. 100, 345–357 10.1016/j.ijfoodmicro.2004.10.030 15854717

[B6] Bockelmann W. Hoppe-Seyler T. (2001). The surface flora of bacterial smear-ripened cheeses from cow's and goat's milk. Int. Dairy J. 11, 307–314 10.1016/S0958-6946(01)00060-7

[B37] Bovill R. Bew J. Baranyi J. (2001). Measurements and predictions of growth for *Listeria monocytogenes* and Salmonella during fluctuating temperature. Int. J. Food Microbiol. 67, 131–137 10.1016/S0168-1605(01)00446-9 11482561

[B38] Castillejo Rodríguez A. M. Barco Alcalá E. García Gimeno R. M. Zurera Cosano G. (2000). Growth modelling of *Listeria monocytogenes* in packaged fresh green asparagus. Food Microbiol. 17, 421–427 10.1006/fmic.1999.0334

[B7] Causton D. R. (1977). A Biologist's Mathematics. London: Edward Arnold

[B8] Chambel L. Sol M. Fernandes I. Barbosa M. Zilhao I. Barata B. (2006). Occurrence and persistence of *Listeria* spp. in environment of ewe and cow's milk cheese diaries in Portugal unveiled by an integrated analysis of identification, typing and spatial – temporal mapping along production cycle. Int. J. Food Microbiol. 116, 52–63 10.1016/j.ijfoodmicro.2006.12.035 17337311

[B9] Dalmasso M. Jordan K. (2014). Absence of growth of *Listeria monocytogenes* in naturally contaminated Cheddar cheese. J. Dairy Res. 81, 46–53 10.1017/S0022029913000678 24345459

[B39] Delignette-Muller M. L. Cornu M. Pouillot R. Denis J.-B. (2006). Use of Bayesian modelling in risk assessment: application to growth of *Listeria monocytogenes* and food flora in cold-smoked salmon. Int. J. Food Microbiol. 106, 195–208 10.1016/j.ijfoodmicro.2005.06.021 16216374

[B10] Denny J. McLauchlin J. (2008). Human *Listeria monocytogenes* infection sin Europe – an opportunity for improved european surveillance. eurosurveillance. Available online at: http://www.eurosurveilance.org/ViewArticle.aspx?Articleid=8082 18445429

[B11] European-Commission. (2005). Commission regulation (EC) No 2073/2005 of 15 November 2005 on microbiological criteria for foodstuffs. Off. J. *L*-338, 1–26 Available online at: http://eur-lex.europa.eu/legal-content/EN/ALL/?uri=CELEX:32005R2073

[B12] Fakruddin M. Mahumder R. M. Bin Mannan K. S. (2011). Predictive microbiology: modeling microbial responses in food. Ceylon J. Sci. 40, 121–131 Available online at: www.pdn.ac.lk/cjsbs/abstract/40.2/4.%2040.2.6%20Microbiology.pdf

[B13] Fox E. Hunt K. O'Brien M. Jordan K. (2011). *Listeria monocytogenes* in Irish farmhouse cheese processing environments. Int. J. Food Microbiol. 145, S39–S45 10.1016/j.ijfoodmicro.2010.10.012 21087802

[B45] Gibson A. M. Bratchell N. Roberts T. A. (1987). The effect of sodium chloride and temperature on the rate and extent of growth of *Clostridium botulinum* type A in pasteurised pork slurry. J. Appl. Bacteriol. 62, 479–490 10.1111/j.1365-2672.1987.tb02680.x 3305458

[B40] Gospavic R. Kreyenschmidt J. Bruckner S. Popov V. Haque N. (2008). Mathematical modelling for predicting the growth of *Pseudomonas* spp. in poultry under variable temperature conditions. Int. J. Food Microbiol. 127, 290–297 10.1016/j.ijfoodmicro.2008.07.022 18775580

[B14] Grijspeerdt K. Vanrolleghem P. (1999). Estimating the parameters of the Baranyi model for bacterial growth. Food Microbiol. 16, 593–605 10.1006/fmic.1999.0285

[B15] Hicks S. J. Lund B. M. (1991). The survival of *Listeria monocytogenes* in cottage cheese. J. Appl. Bacteriol. 70, 308–314 10.1111/j.1365-2672.1991.tb02941.x 1905281

[B16] Jason A. C. (1983). A deterministic model for monophasic growth of batch cultures of bacteria. Antonie Van Leewenhoek 49, 513–536 10.1007/BF00399845 6370130

[B17] Liu S. Puri V. M. (2008). Dynamic growth models for *L. monocytogenes* during ripening in Camembert cheese. Food Sci. Technol. 41, 511–520 Available online at: http://www.sciencedirect.com/science/article/pii/S0023643807001259

[B41] McMeekin T. A. Olley J. N. Ross T. Ratkowsky D. A. (1993). Predictive Microbiology: Theory and Application. Taunton, UK: Research Studies Press Ltd

[B50] McMeekin T. A. Ross T. (2002). Predictive microbiology: providing a knowledge-based framework for change management. Int. J. Food Microbiol. 78, 133–153 10.1016/S0168-1605(02)00231-3 12222630

[B19] Morgan F. Bonnin V. Mallereau M.-P. Perrin G. (2001). Survival of *Listeria monocytogenes* during manufacture, ripening, and storage of soft lactic cheese made from raw goat milk. Int. J. Food Microbiol. 64, 217–221 10.1016/S0168-1605(00)00452-9 11252508

[B20] Murphy P. M. Rea M. C. Harrington D. (1995). Development of a predictive model for growth of *Listeria monocytogenes* in a skim milk medium and validation studies in a range of dairy products. J. Appl. Bacteriol. 80, 557–565 10.1111/j.1365-2672.1996.tb03257.x 9072528

[B42] Pouillot R. Albert I. Cornu M. Denis J.-B. (2003). Estimation of uncertainty and variability in bacterial growth using Bayesian inference. Application to *Listeria monocytogenes*. Int. J. Food Microbiol. 81, 87–104 10.1016/S0168-1605(02)00192-7 12457583

[B21] Presser K. A. Ratkowsky D. A. Ross T. (1997). Modeling the growth rate of *Escherichia coli* as a function of pH and lactic acid concentration. Appl. Environ. Microbiol. 63, 2355–2360 9172355 10.1128/aem.63.6.2355-2360.1997 PMC168528

[B22] Ratkowsky D. A. Olley J. McMeekin T. A. Ball A. (1982). Relationship between temperature and growth rate of bacterial cultures. J. Bacteriol. 149, 1–5 7054139 10.1128/jb.149.1.1-5.1982 PMC216584

[B23] Rosso L. Lobry J. R. Bajard S. Flandrois J. P. (1995). Convenient model to describe the combined effects of temperature and pH on microbial growth. Appl. Environ. Microbiol. 61, 610–616 16534932 10.1128/aem.61.2.610-616.1995 PMC1388350

[B24] Rudolf M. Scherer S. (2001). High incidence of *Listeria monocytogenes* in European red smear cheese. Int. J. Food Microbiol. 63, 91–98 10.1016/S0168-1605(00)00413-X 11205958

[B25] Ryser E. T. Marth E. H. (1987). Fate of *Listeria monocytogenes* during the manufacture and ripening of camembert cheese. J. Food Prot. 50, 372–378 30965516 10.4315/0362-028X-50.5.372

[B26] Scallan E. Hoekstra R. M. Angulo F. J. Tauxe R. V. Widdowson M. A. Roy S. L. (2011). Foodborne illness acquired in the United States–major pathogens. Emerg. Infect. Dis. 17, 7–15 10.3201/eid1701.P11101 21192848 PMC3375761

[B27] Schvartzman M. S. Belessi X. Butler F. Skandamis P. Jordan K. (2010). Comparison of growth limits of *Listeria monocytogenes* in milk, broth, and cheese. J. Appl. Microbiol. 109, 1790–1799 10.1111/j.1365-2672.2010.04807.x 20649836

[B28] Silva I. M. M. Almeida R. C. C. Alves M. A. O. Aleida P. F. (2002). Occurrence of *Listeria* spp. in critical control points and the environment of Minas Frescal cheese processing environment. Int. J. Food Microbiol. 81, 241–248 10.1016/S0168-1605(02)00223-4 12485750

[B43] Spor A. Dillmann C. Wang S. de Vienne D. Sicard D. Parent E. (2010). Hierarchical Bayesian modelling for *Saccharomyces cerevisiae* population dynamics. Int. J. Food Microbiol. 142, 25–35 10.1016/j.ijfoodmicro.2010.05.012 20576304

[B29] Swaminathan B. Gerner-Smidt P. (2007). The epidemiology of human listeriosis. Microb. Inf. 9, 1236–1243 10.1016/j.micinf.2007.05.011 17720602

[B30] Tenenhaus-Aziza F. Daudin J.-J. Maffre A. Sanaa M. (2013). Risk based approach for microbiological food safety management in the dairy industry: the case of *Listeria monocytogenes* in soft cheese made from pasteurised milk. Risk Anal. 34, 56–74 10.1111/risa.12074 23777564

[B31] Tiengenun S. Ratkowsky D. A. McMeekin T. A. Ross T. (2000). Growth limits of *Listeria monocytogenes* as a function of temperature, pH, NaCl and lactic acid. Appl. Environ. Microbiol. 66, 4979–4987 10.1128/AEM.66.11.4979-4987.2000 11055952 PMC92408

[B32] Vose D. (2008). Risk Analysis: A Quantitative Guide. Chichester: John Wiley & Sons

[B33] Xiong R. Xie G. Edmondson A. S. Linton R. H. Sheard M. A. (1999). Comparison of the Baranyi model with the modified Gompertz equation for modeling thermal inactivation of *Listeria monocytogenes* Scott A. Food Microbiol. 6, 269–279 10.1006/fmic.1998.0243

[B34] Zwietering M. H. Jongenburger I. Rombouts F. M. van T. Riet K. (1991). Modeling of the bacterial growth curve. Appl. Environ. Microbiol. 57, 1875–1881 16348228 10.1128/aem.56.6.1875-1881.1990 PMC184525

